# Transcriptomic profiling revealed important roles of amino acid metabolism in fruiting body formation at different ripening times in *Hypsizygus marmoreus*

**DOI:** 10.3389/fmicb.2023.1169881

**Published:** 2023-04-25

**Authors:** Quanju Xiang, Muhammad Arshad, Yakun Li, Huijuan Zhang, Yunfu Gu, Xiumei Yu, Ke Zhao, Menggen Ma, Lingzi Zhang, Maolan He, Qiang Chen

**Affiliations:** ^1^College of Resources, Sichuan Agricultural University, Chengdu, Sichuan, China; ^2^Qinghai Spring Medicinal Resources Technology Co., Ltd., Chengdu, Sichuan, China

**Keywords:** differentially expressed genes, transcriptomics, amino acid metabolism, enzymes, lignocellulose

## Abstract

**Introduction:**

Hypsizygus marmoreus is an industrial mushroom that is widely cultivated in East Asia. Its long postripening stage before fruiting severely limits its industrialized production.

**Methods:**

Five different mycelial ripening times (30, 50, 70, 90, and 100 d) were chosen and primordia (30P, 50P, 70P, 90P, and 110P) were collected for comparative transcriptomic analyses. The corresponding substrates (30F, 50F, 70F, 90F, and 110F) were used for nutrient content and enzyme activity determination.

**Results:**

In pairwise comparisons between 110P and other primordia, a total of 1,194, 977, 773, and 697 differentially expressed genes (DEGs) were identified in 30P_110P, 50P_110P, 70P_110P, and 90P_110P, respectively. Gene Ontology (GO) and Kyoto Encyclopedia of Genes Genomes (KEGG) functional enrichment analyses revealed that the DEGs were mainly associated with amino acid metabolism, and lipid and carbohydrate metabolism pathways. Tyrosine, tryptophan, phenylalanine and histidine metabolism were enriched in all groups. Among the main carbon nutrients, the contents of cellulose and hemicellulose were high, and the lignin content decreased with the extension of the ripening time. Laccase had the highest activity, and acid protease activity decreased with the extension of the ripening time.

**Discussion:**

The highly enrichment for amino acid metabolic pathways in primordia reveals that these pathways are essential for fruiting body formation in H. marmoreus, and these results will provide a basis for the optimization of its cultivation.

## 1. Introduction

Mushrooms, which belong to higher or macro-fungi (Basidiomycetes) differ from lower fungi, such as unicellular fungi yeast or filamentous fungi (e.g., *Aspergillus fumigatus*) in morphological structure. The life cycles of most fungal taxa generally involve different developmental stages or morphotypes, including the germination of spores, growth of the mycelial, and formation of fruiting bodies (Willetts, 1972). The growth of the mycelium referred to as vegetative growth, is important for nutrient absorption. After a certain stage, mycelia begin to differentiate and transform from vegetative to reproductive growth under certain conditions. Environmental factors, such as light (Jang et al., 2013), temperature, CO_2_ concentration, and humidity, as well as biological factors (Sun et al., 2020) play critical roles in mushroom growth and development. Some functional genes, such as *Noxs* (Mu et al., 2014), *dst* (Terashima et al., 2005), and *Ubc2* (Nakazawa et al., 2011), transcription factors, such as WC-1 (Idnurm and Heitman, 2005) and FlbB (Etxebeste et al., 2010), and signaling pathways, such as mitogen-activated protein kinase (MAPK) (Park et al., 2008), are reported to be involved in the regulation of sexual development. However, the molecular mechanisms underlying the transformation from vegetative growth to reproductive growth are still unclear.

*Hypsizygus marmoreus* is well known for its nutritional value and remarkable flavor (Harada et al., 2003). Its active compounds, such as glycoprotein, also have anti-tumor and anti-inflammatory effects (Lam and Ng, 2001; Tsai et al., 2013; Chien et al., 2016). Studies of *H. marmoreus* have mainly focused on its cultivation, nutritional properties, and bioactive compounds (Kohsuke et al., 2018; Mleczek et al., 2018; Oliveira et al., 2019; Tsai et al., 2019), and several recent studies have evaluated its growth and development (Yang et al., 2021). The genome of *H. marmoreus* was assembled and annotated (Min et al., 2018), and an efficient *Agrobacterium*-mediated transformation method was developed (Zhang et al., 2014), providing a basis for detailed molecular analyses. Differentially expressed genes (DEGs) from four transcriptomes of *H. marmoreus* suggested that light and nitrogen metabolism affect fruiting body initiation (Zhang et al., 2015). A comparative proteomic analysis revealed important roles of proteins involved in biomass increase, cell proliferation, signal response, and differentiation in the primordia and unmatured fruiting bodies of *H. marmoreus* (Yang et al., 2021). Additional studies have evaluated the molecular mechanism underlying the response to environmental changes. The mycelia of *H. marmoreus* undergoes autolysis and apoptosis in response to heat stress (Xu et al., 2021). These studies provide meaningful information about the growth and development of *H. marmoreus*.

Compared with other industrially cultivated mushrooms, such as *Agaricus bisporus*, *Pleurotus eryngii*, and *Flammulina filiformis*, mycelial growth for an additional 60 days (postripening) is required for high-quality agronomic traits and yields of *H. marmoreus*, requiring more time (about 110 to 120 days) from inoculation to harvest (Lam and Ng, 2001). The long cultivation period provides an opportunity for contamination and severely limits industrialized production. Multi-omics analysis has shown that the contents of arginine and citrate in the substrate are key factors in the postripening period (Gong et al., 2022). However, it is not clear why *H. marmoreus* requires such a long postripening time; thus, it is essential to understand the life cycle of this mushroom. The vegetative mycelium mainly functions in nutrient absorption, and the nutrient depletion is thought to promote fruiting differentiation. This theory was supported by the finding that sufficient nutrients will maintain the *Saprolegnia* in the vegetative growth stage, and only when nutrients became poor, does the species shift to reproductive growth (Klebs, 1899). In addition, a positive correlation between the length of the culture bag and vegetative growth period of the mycelium was observed, indicating nutrient depletion was faster in shorter culture bags contained less nutrient, and make it become nutrient depleted faster (Jin et al., 2014).

Research on the developmental mechanisms of the *H. marmoreus* fruiting body is limited, especially research on the influence of the postripening time on fruiting. To better understand the molecular mechanism underlying the postripening time on fruiting body formation, a transcriptome analysis of primordia from *H. marmoreus* five postripening times was performed using Illumina technology to identify DEGs. Additionally, nutrient contents and their degrading enzyme activity levels were also determined. These results provide insight into the long postripening time and improve our understanding of molecular mechanisms underlying fruiting body development in *H. marmoreus*.

## 2. Materials and methods

### 2.1. Sample preparation

The *H. marmoreus* strain YF2018557 was first cultivated in liquid spawn developed using PDA (potato 200 g kg^–1^, glucose 20 g kg^–1^ and 1% peptone) in 6 days at 200 rpm and 21°C. The substrate medium (C:N = 27:1) was prepared by using 51% cotton seed shell, 20% bran, 20% wood chips (natural fermentation for 6 months), 5% cornmeal, 3% soybean meal, 1% lime and 65% water content. A total of 800 g dry solid substrate per bag was prepared and sterilized (121°C, 2 h). Then, 25 ml of the liquid spawn was inoculated into to the cultivation bag and kept at 23°C and 70% humidity. The bags were cultivated for 30, 50, 70, 90, 100, 105, and 110 days and transferred to the fruiting room (16°C and 95% humidity). Five primordia (30, 50, 70, 90, and 110P) were collected from samples at 30, 50, 70, 90, and 110 days and immediately frozen in liquid nitrogen for RNA extraction. The corresponding substrates (30, 50, 70, 90, and 110F) were collected and kept at 4°C for nutrient and enzyme determination. Four samples (90, 100, 105, and 110) continued fruiting for analyses of morphology and yield.

### 2.2. RNA extraction, cDNA library construction and Illumina sequencing

Five cDNA libraries were prepared for five different time points (30, 50, 70, 90 and 110 days) and were separately sequenced by using the Illumina platform. Total RNAs from all samples were extracted by using TRIzol reagent (Takara, Kusatsu, Japan). RNA quality and quantity were observed by using a spectrophotometer and a 2100 Bioanalyzer. The mRNAs with polyA structures were enriched using magnetic beads were bound to Oligo d(T). Using mRNA as the template, the cDNA was synthesized by using NEBNext Ultra II RNA Library Prep Kit for Illumina (NEB, NE, USA). After library construction was completed, library fragment enrichment was carried out by PCR amplification, followed by size selection, and the library size was 450 bp. After quantification, paired-end libraries were sequenced using the Illumina HiSeq 2000 (2 bp × 150 bp read length). The data presented in this study are deposited in the NCBI’s BioProject repository, accession number “PRJNA906646”.

### 2.3. Analysis and functional annotation of differentially expressed genes

HTSeq was used to compare the read counts, defined as the original expression levels, normalized by fragments per kilo bases per million fragments (FPKM) (Mortazavi et al., 2008). DESeq was used to identify DEGs with the following thresholds for significance: | log2FoldChange| > 1 and *p* < 0.05. Gene ontology (GO) and kyoto encyclopedia of genes and genomes (KEGG) functional enrichment analyses were carried out using Goatools^1^ and KOBAS^2^ (Chen et al., 2011).

### 2.4. Real-time quantitative PCR analysis

A qRT-PCR assay was performed to confirm the RNA-seq results. Reverse transcription of 1.5 μg of total RNA into cDNA was conducted by following the instructions provided with the Reverse Transcription Kit (Shenggong, Shanghai, China). qRT-PCR primers were designed using the Primer-Blast.^3^ qRT-PCR was performed using SYBR Green and the iQ5 thermo cycler (Bio-Rad, Hercules, USA). Each biological sample was amplified in three technical replicates. Each reaction system consisted of 1 ml of 10-fold diluted cDNA, 0.5 ml of each primer (1 μmol/L), 10 ml of ChamQ Universal SYBR Qpcr Master Mix (Vazyme, Nanjing, China), and nuclease-free water to a final volume of 20 ml. The 28S gene (gene id: RHE_CH00059) from *H. marmoreus* was used as an endogenous control. PCR conditions were as follows: 95°C for 5 min, 1 cycle; followed by 40 cycles at 95°C for 15 s, 60°C for 15 s, and 72°C for 30 s. A dissociation curve was generated to ensure specificity.

### 2.5. Enzyme determinations

Twenty grams of substrate were dissolved in 100 ml of citrate–phosphate buffer (50 mm, Ph 7.0) at 25°C and 100 rpm for 3 h. After standing for 1 min, the samples were centrifuged at 7,000 rpm for 20 min. The supernatant was filtered through a filter paper (Whatman, Metestone, UK) under vacuum and then used for enzyme determination.

Laccase (Lac) activity was determined by the Bourbonnais (Bourbonnais et al., 1995). The 3 ml enzyme reaction system contained 2.7 ml of 0.1 mol/L acetic acid-sodium acetate buffer (Ph = 4.5), 0.1 ml of crude enzyme solution and 0.2 ml of 0.5 mmol/L ABTS. The reaction was carried out at 25°C for 3 min, and the change in absorbance at 420 nm was measured. The amount of enzyme required to oxidize 1 μmol ABTS/min at a reaction temperature of 25°C was defined as an enzyme activity unit (U).

Manganese peroxidase (Mnp) activity was measured with phenol red as the substrate (Kuwahara et al., 1984). The reaction mixture contained 0.5 ml of crude enzyme solution, 0.1 ml of phenol red solution (0.1%), 0.1 ml of sodium lactate Ph 4.5 (250 mm), 0.2 ml of bovine serum albumin solution (0.5%), 0.05 ml of manganese sulfate (2 mm), and 0.05 ml of H_2_O_2_ (2 mm) in sodium succinate buffer (20 mm, Ph 4.5). Activity was determined as the increase in absorbance at 610 nm per minute per milliliter. One unit of enzyme activity was defined as the amount of enzyme oxidizing 1 μmol of substrate per minute. The enzyme activity was expressed as U/L.

Lignin peroxidase (Lip) activity assay was performed by reference to the method of Tien and Kirk (1984). The 2.5 ml reaction system contained 575 ml (9.12 mmol/L) resveratrol, 940 ml of tartaric acid buffer (130 mmol/L, Ph 2.5), 910 Ul of H_2_O and 40 ml of H_2_O_2_ (0.3%). The reaction was carried out at 25°C for 3 min and activity was measured at 310 nm. The amount of enzyme required to oxidize 1 μmol resveratrol per minute at 25°C was defined as an enzyme activity unit (U), and the activity of the enzyme per mg is a specific activity unit.

Cellulase, α-amylase and acid protease activity assays were performed using spectrometric kits obtained from Suzhou Keming Biotechnology Co., Ltd.^4^ The spectrophotometer was preheated for 30 min, and the wavelength was adjusted to 620 nm, 540 nm and 680 nm for cellulase, α-amylase and acid protease, respectively. Distilled water was used as a control and the enzyme activity content was determined.

### 2.6. Lignin, cellulose, and hemicellulose content determination

Lignin, cellulose, and hemicellulose assays were performed using the spectrometric kits obtained from Suzhou Keming Biotechnology Co., Ltd. (see text footnote 4) The spectrophotometer used for nutrient determination was preheated for 30 min, and absorbance was adjusted to 280 nm for lignin, 620 nm for cellulose and 540 nm for hemicellulose. Distilled water was used as a control and nutrient contents were determined.

### 2.7. Statistical analysis

Data obtained from triplicate experiments are expressed as means ± standard deviation (SD). Statistical Product and Service Solutions (SPSS, version 17.0, Chicago, IL, USA) was used for data analysis. Means were compared using the least significant difference (LSD) test at *p* ≤ 0.05. Pearson’s correlation coefficients were determined using SPSS to evaluate correlations between the polysaccharide content and enzyme activity levels.

## 3. Results

### 3.1. Fruiting traits for different post-ripening times

The mycelium of *H. marmoreus* needs to grow for 110 days to ensure its quality and yield. The 90-day sample had a shorter stipe (by 1 cm) and lower yield (by 59 g) than those of the 110-day sample (Supplementary Figure 1, Table 1).

**TABLE 1:** Stipe length and yield of *Hypsizygus marmoreus* for four different mycelial ripening times.

Time (d)	Stipe length (cm)	Product per bag (g)
90	13.0–14.0	459.7 ± 7.0c
100	13.5–15.0	504.3 ± 5.7b
105	14.0–15.0	503.0 ± 5.0b
110	14.0–15.5	518.3 ± 6.5a

Values are the means of three replicates ± SD. Different letters next to the numbers indicate statistical differences (*p* < 0.05) according to ANOVA-LSD test.

### 3.2. RNA sequencing and transcriptome analysis

**FIGURE 1:**
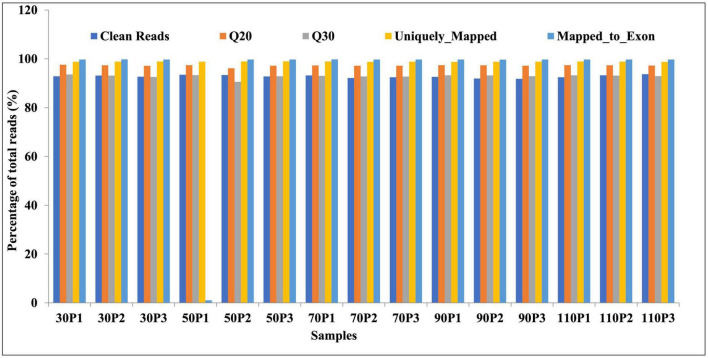
Sequencing quality analysis.

To investigate the molecular basis of ripening time on fruiting-body formation, we performed RNA-seq analyses of primordia (30, 50, 70, 90, and 110P) obtained from five ripening times: 30, 50, 70, 90, and 110 days. Three biological replicates were set for each sample. Raw reads of the transcriptome project have been deposited in NCBI’s BioProject accession number PRJNA906646. A total of 15 high-quality RNA libraries were constructed. Paired-end sequencing was performed using the Illumina HiSeq 4000 platform, followed by the removal of the raw reads containing adapters and low-quality reads. A total of 55.5 GB of high-quality clean reads was obtained, and the percentages of Q20 bases and Q30 bases were 97.2 and 92.9%, respectively (Figure 1). For all libraries, approximately 98.8 and 93.1% of the reads were mapped to the *H. marmoreus* reference genome and exons (Figure 1). Gene expression values were normalized as FPKM. Taken together, our sequencing data were of high quality and displayed good correlations among biological replicates and thus could be used for subsequent analyses.

### 3.3. Identification of differentially expressed genes

**FIGURE 2:**
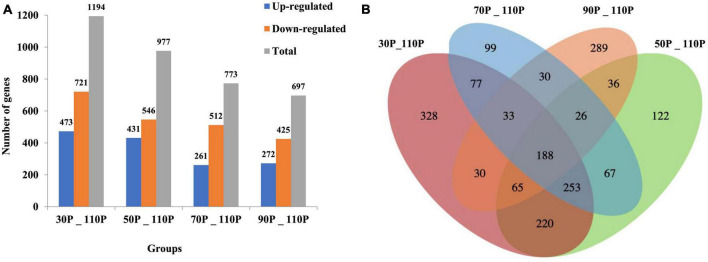
Differentially expressed genes shown by a bar plot **(A)** and Venn diagram **(B)**. The histogram shows the numbers of up- and down-regulated differentially expressed genes (DEGs) in each group pairwise comparison (30P_110P, 50P_110P, 70P_110P, and 90P_110P) of *Hypsizygus marmoreus*. **(B)** Venn diagram showing the overlap in DEGs between four comparisons.

A comparative transcriptome analysis was performed between samples with ripening times of 30-, 50-, 70-, 90 and 110 days (normally used in industrial production). DESeq was used to identify DEGs, with threshold values of log2FoldChange > 1 and *p* < 0.05. A total of 1,194, 977, 773, and 697 DEGs were identified in 30P_110P, 50P_110P, 70P_110P, and 90P_110P, respectively (Figure 2, Supplementary Table 1).

### 3.4. Functional annotation of differentially expressed genes

**FIGURE 3:**
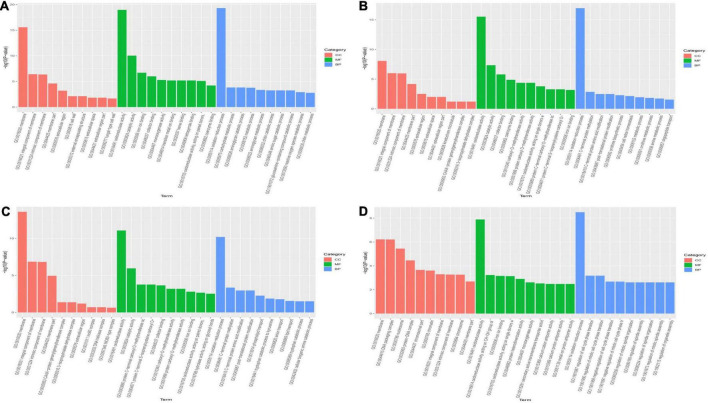
Gene ontology (GO) enrichment analysis of differentially expressed genes in each group. The *Y*-axis represents the -log10 (*p*-value) and *X*-axis represents the GO functional classification based on biological process, cellular component, and molecular function. **(A)** 30P_110P, **(B)** 50P_110P, **(C)** 70P_110P, **(D)** 90P_110P. BP, biological processes; CC, cellular components; MF, molecular functions.

To better understand the functions of DEGs involved in primordium differentiation after different ripening times, all DEGs were subjected to a GO enrichment analysis. The enriched terms were grouped into three functional categories: biological processes, cellular components, and molecular functions (Supplementary Table 2). Figure 2A shows the top 30 enriched GO terms enriched in each category (10 terms per functional category, *p* < 0.05). The significantly enriched biological process terms (*p* < 0.05) were associated with growth and development, with “oxidation reduction process” (GO: 0055114) being the most highly enriched (Figure 3). In the molecular function category, DEGs were involved in oxidoreductase activity (GO: 0016491). In the cellular component category, a large number of DEGs were assigned to the membrane (GO: 0016020) component. Based on the rich factor, False discovery rate (FDR) values, and number of genes, catalytic activity and membrane were the most enriched terms in all groups.

**FIGURE 4:**
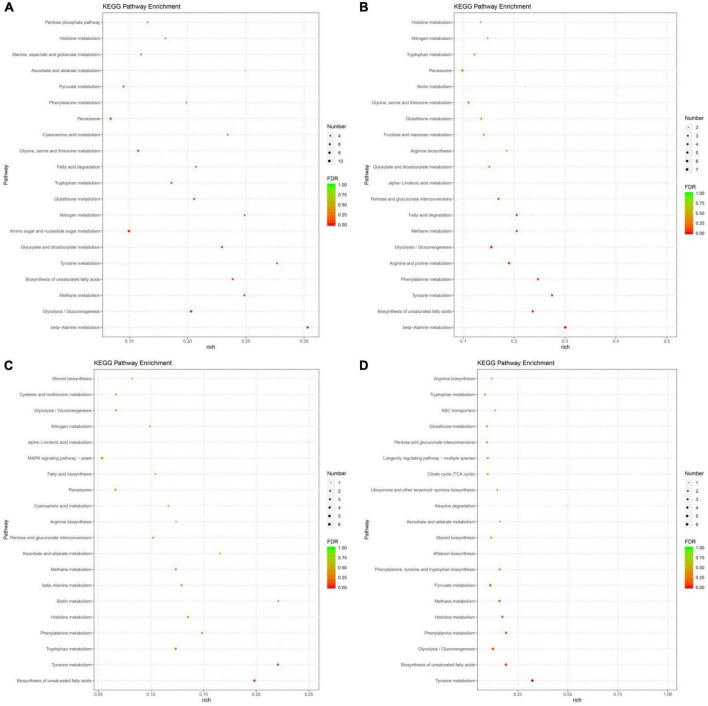
The most significantly-enriched kyoto encyclopedia of genes and genomes (KEGG) pathway of differentially expressed genes (DEGs) in 30P_110P **(A)**, 50P_110P **(B)**, 70P_110P **(C)**, and 90P_110P **(D)**.

Furthermore, a KEGG analysis was used to classify the functions of annotated DEGs. Significant enriched pathways are shown in Supplementary Table 3. Based on the pathway enrichment analysis, DEGs were involved in metabolism, including amino acid, carbohydrate, nitrogen and fatty acid metabolism (Figure 4). In addition to pathways related to nutrient metabolism, pathways related to signaling, such as mitogen-activated protein kinase (MAPK) pathway, and transporter were also enriched.

**FIGURE 5:**
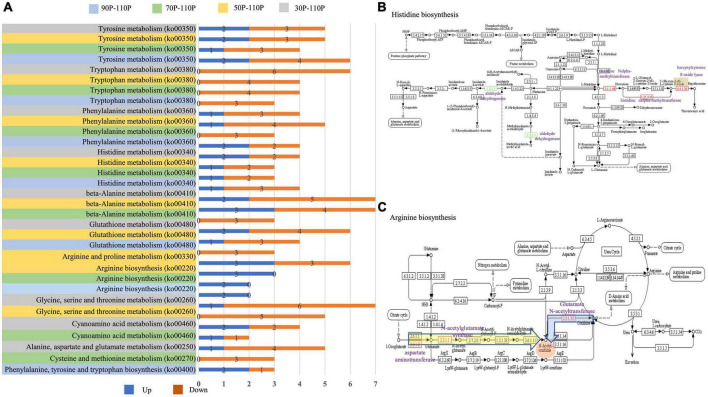
Amino acid pathways of differentially expressed genes (DEGs) annotated by kyoto encyclopedia of genes and genomes (KEGG). **(A)** Significantly enriched amino acid pathways in the top 20 KEGG pathways. The up-regulated and down-regulated genes are shown by the blue and orange bar, respectively. The specific values are marked on the side. Amino acid metabolism pathway are displayed on the left: gray indicates 30P_110P; yellow 50P_110P; green 70P_110P and blue 90P_110P. **(B)** Histidine metabolism (http://www.kegg.jp/kegg-bin/show_pathway?map00340) identified by KEGG annotation. The down-regulated genes are shown in green, and up-regulated are shown in red. One specific up-regulated gene in 30P_110P is marked in orange. The names of DEGs are marked in purple. **(C)** Arginine metabolism (https://www.kegg.jp/kegg-bin/show_pathway?map00220) identified by KEGG annotation. DEGs identified in all three groups are marked in a yellow box, and one gene specifically identified in 50P_110P is marked in a blue box. The red box indicates the intermediate product, N-acetyl-ornithine. The names of DEGs are shown in purple.

In the top 20 KEGG pathways, nine, seven, seven and six amino acid metabolism pathways were identified in 30P_110P, 50P_110P, 70P_110P, and 90P_110P, respectively (Figure 5A). Tyrosine, tryptophan, phenylalanine and histidine metabolism were enriched in all four groups, while beta-alanine and glutathione metabolism were enriched in 30P_110P, 50P_110P and 70P_110P. Arginine metabolism was enriched in the later three groups: 50P_110P, 70P_110P and 90P_110P. All DEGs in the tryptophan metabolism pathway were down-regulated, while all DEGs in arginine metabolism pathway were up-regulated. The two down-regulated genes involved in histidine metabolism encoded alcohol dehydrogenase (Figure 5B). The up-regulated genes related to arginine metabolism involved in the biosynthesis of intermediate product N-acetyl-ornithine (Figure 5C).

### 3.5. Validation of DEGs by qRT-PCR

**FIGURE 6:**
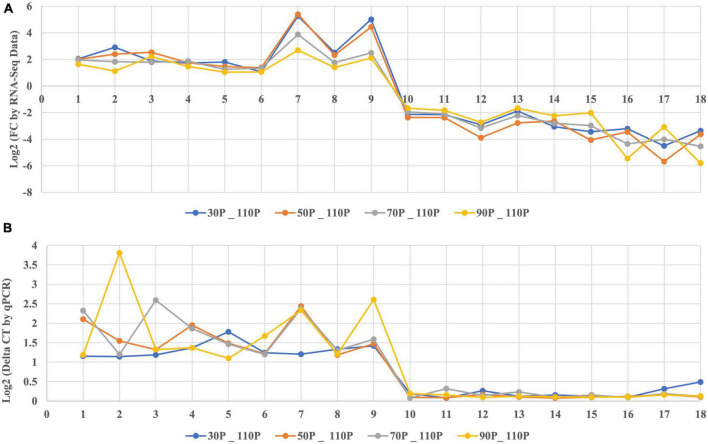
Quantitative RT-PCR analysis of selected genes. **(A)** Log2 fold change determined by RNA-seq, **(B)** Log2 fold change determined by qRT-PCR. Different colors represent the fold changes from different samples: blue, 30P_110P, orange, 50P_110P, gray, 70P_110P, yellow, 90P_11P. Numbers 1 to 18 represent genes *Hypma_015692*, *Hypma_006925*, *Hypma_006711*, *Hypma_013322*, *Hypma_015185*, *Hypma_002213*, *Hypma_013537*, *Hypma_001039*, *Hypma_012178*, *Hypma_011936*, *Hypma_012533*, *Hypma_005024*, *Hypma_014954*, *Hypma_012995*, *Hypma_003453*, *Hypma_010797*, *Hypma_014463*, and *Hypma_011943*, respectively.

To evaluate the reliability and reproducibility of the transcriptome data, we used the same RNA-Seq samples for qRT-PCR. A total of 18 genes were selected for qRT-PCR, including 9 up-regulated and 9 down-regulated genes representing different KEGG subcategories. By a correlation analysis of the two sets of data, the fold change values of genes obtained by qRT-PCR were compared to those obtained by RNA-Seq. The relative trends in expression based on qRT-PCR were consistent with those for RNA-Seq data (Figure 6).

### 3.6. Enzyme activity levels

The main function of vegetative growth is to absorb nutrients to meet the needs of fruiting body formation and growth. Therefore, the activity levels of nutrient-degrading enzymes were measured in 30, 50, 70, 90, and 110S.

**FIGURE 7:**
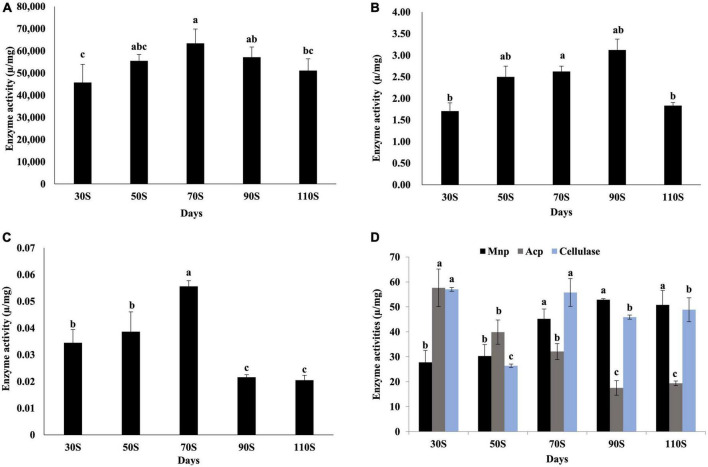
Activity levels of enzymes involved in nutrient degradation. **(A)** Laccase, **(B)** lignin peroxidase, **(C)** α-amylase, **(D)** manganese peroxidase, acid protease, and cellulase values are the means of three replicates ± SD. Different letters above columns indicate statistically significant differences between individual enzyme activities at *p* < 0.05 (ANOVA-LSD, *n* = 3).

Among the four main lignocellulose degradation enzymes (lignin peroxidase, Lip; manganese peroxidase, Mnp; laccase, Lac, and cellulase), activity levels were highest for laccase, followed by cellulase and Mnp, and Lip showed very low enzyme activities (Figure 7), suggesting the laccase is the main enzyme for lignin degradation in *H. marmoreus*. The activity of laccase increased continuously from 30 to 70S and reached a maximum value of 6.3 × 10^4^ U/mg (Figure 7A). Cellulase activity reached a peak in 30S (57.0 U/mg) and 70S (55.9 U/mg) samples (Figure 7D). The activity of Mnp increased with the extension of the culture time, and with a maximum value (52.9 U/mg) in the 90S sample (Figure 7D). Enzymes involved in starch and protein degradation were also evaluated. Acid protease (ACP) showed highest activity (57.6 U/mg) in the 30S sample and decreased with increasing time, indicating that protein degradation occurred at early culture stages. The α-amylase activity levels were low in all samples (Figure 7C).

### 3.7. Lignocellulose contents for different ripening times

**FIGURE 8:**
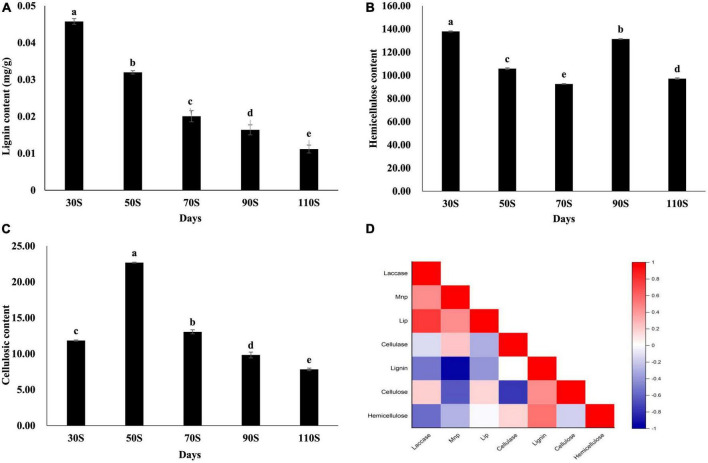
Contents of lignin **(A)**, cellulose **(B)**, hemicellulose **(C)**, and results of correlation analyses **(D)**. Pearson correlations coefficients were evaluated between contents and enzyme activities. Red represents a positive correlation, and blue represents a negative correlation (*p* < 0.05). The intensity of the color represents the significance level. Values are the means of three replicates ± SD. Different letters above columns indicate statistically significant differences between individual nutrient content at *p* < 0.05 (ANOVA-LSD, *n* = 3).

Lignocellulose is the main nutrient in the substrate, and the contents of lignin, cellulose, and hemicellulose were determined under different ripening time. The substrate has the highest hemicellulose content and the lowest lignin content (Figure 8). The lignin content continued to decrease with the extension of the cultivation time, from 0.045 (30S) to 0.010 mg/g (110S) (Figure 8A). The highest cellulose content was detected in the 50S sample (22.675 mg/g) and dropped as the culture time increased (Figure 8B). The content of hemicellulose fluctuated, showing relatively high contents in 30 and 90S samples (Figure 8C).

The correlation analysis revealed that the cellulose content was significantly correlated with laccase and Lip levels, and the hemicellulose content was significantly correlated with cellulose levels (Figure 8D).

## 4. Discussion

*Hypsizygus marmoreus* is a popular edible mushroom and analyses of on the cultivation practices are critical to improve its quality and quantity. Its long cultivation period and yield have been important focuses of research. To understand the long postripening period in *H. marmoreus*, a comparative transcriptomics analysis of primordia obtained after different ripening times was performed. The greater the difference in ripening time, the greater the number of DEGs, indicating that mycelial ripening time influences fruiting body development in *H. marmoreus*.

Amino acids not only serve as building blocks of proteins, polypeptides, and other nitrogenous biomolecules in microorganisms, but also play important roles in metabolism, survival, inter-specific interactions, and virulence (Wilson et al., 2019). Amino acid levels are an important environmental cue affecting the sexual development of Ascomycetes, and are an enriched process within the fruiting body in *Tricholoma matsutake* (Liu et al., 2021). In comparison between 110P and other samples, DEGs were enriched in amino acid metabolism pathways. Tryptophan, tyrosine, phenylalanine and histidine metabolic pathway were enriched in four groups. Tyrosine, phenylalanine, and tryptophan are aromatic amino acids that share the same precursor for the synthesis of phosphoribosyl. Whether phosphoribosyl is a key signaling factor for the mature mycelial needs further research. The content of arginine in the substrate of *H. marmoreus* has been reported to increase as the mycelium culture time increases, and the addition of arginine shortens the postripening period and increases the fruiting body yield (Gong et al., 2022). The external addition of arginine influences the rate of development *Sordaria macrospora* Auersw (Molowitz and Hock, 1976). Arginine metabolism was enriched in DEGs between 50 and 110P, 70 and 110P, and 90 and 110P in this study. The expression levels of two genes (*Hypma_015692* and *Hypma_001474*) involved in arginine metabolism were up-regulated in all three groups; these two genes are involved in the synthesis of the intermediate N-acetyl-ornithine, indicating that the synthesis of N-acetyl-ornithine tended to decrease with the extension of the ripening time. Another gene (*Hypma_002214*) involved in N-acetyl-ornithine synthesis was up-regulated in 50P_110P. Acetyl-ornithine is usually converted to ornithine in arginine metabolism, which is mainly involved in the urea cycle and plays an important role in the excretion of ammonia nitrogen. The up-regulation of these genes implied that amino acid metabolism may be strong during the early mycelial ripening period, and the urea cycle eliminates ammonia, a by-product of amino acid metabolism, reducing ammonia toxicity.

Amino acids, normally obtained from proteins in the culture substrate via proteinases, are translocated into the vegetative mycelium and utilized for the growth of the fruiting body (Terashita et al., 1998). A metal proteinase inhibitor could completely inhibit the fruiting body formation in *H. marmoreus* [36]. Although amino acid components were not determined in this study, activity of ACP gradually decreased with the extension of the ripening time, indicating that there is a protein deficiency at a later ripening period.

A nitrogen shortage in the mycorrhizosphere stimulates the establishment of the ectomycorrhizal symbiosis (Lengeler et al., 2000; Bidartondo, 2001). In Basidiomycetes, low levels of nitrogen may be commercially beneficial because this condition promotes fruiting. Nitrogen metabolism is an important factor in the regulating of morphogenesis in *A. bisporus* and for promoting fruit body maturation in *H. marmoreus* (Eastwood et al., 2013). Two genes encoding ammonium transporter (*Hypma_001598* and *Hypma_015145*) and nitrate reductase (*Hypma_005776*) were up-regulated in 30P_110P, indicating that with the extension of the ripening time, nitrogen nutrition gradually decreased, thereby inducing the expression of related genes; this nitrogen starvation might be a key factor promoting fruit body development. Peroxisome is involved in the selective degradation of individual cytosolic proteins and oxidization of D-amino acids in eukaryotes (Finley, 2009). Compared to 110P, there are eight, six, four and two up-regulated genes in the peroxisome pathway in the 30, 50, 70, and 90P groups. The number of DEGs in the peroxisome pathway decreased as the ripening time increased, suggesting that the nutrient starvation in the later culture stage may induce protein degradation.

Carbohydrates are the main nutrients for the growth of macro-fungi. Various enzymes are involved in the degradation of lignocellulose, with significant roles in morphological changes and nutrient absorption. Laccase is involved in the degradation of lignin (Gutiérrez et al., 2012), development of fruiting-bodies, pigmentation, and other processes. *H. marmoreus* is a highly potential laccase producer (Galic et al., 2020). Compared to levels of 50 and 70P, a laccase gene (*Hypma_012153*) was up-regulated by about 14.49- and 25.87-fold in 110P. The gene may play important roles in the process of hyphal maturation. The degradation of cellulose and hemicellulose is closely dependent on the extent of lignin removal. The lignin content was relatively low, while the cellulose and hemicellulose content were high, which may be due to the biodegradation of lignin in the early stage (from inoculation to 30 days). Amylase may play important roles in mycelial maturation and fruit body growth in *H. marmoreus*; its extracellular activity was low, while its intracellular activity was relatively high (Terashita et al., 1998). The activity of amylase (extracellular) was relatively low.

## 5. Conclusion

The highly enrichment for amino acid metabolic pathways in primordia reveals that these pathways are essential for fruiting body formation in *H. marmoreus*. Some components of amino acid metabolic pathways, especially in tryptophan, tyrosine, phenylalanine, histidine, and arginine pathways, may be key factors affecting postripening. With the extension of the ripening time, the lignin content gradually decreased, while the cellulose and hemicellulose contents increased. Laccase is the main enzyme involved in lignin degradation in *H. marmoreus*. Further research on the long postripening time should focus on factors contributing to amino acid metabolism. Our results contribute to a better understanding of mechanisms underlying *H. marmoreus* growth and development, and highlights the promising application of amino acid accumulation in high-efficiency cultivation.

## Data availability statement

The datasets presented in this study can be found in online repositories. The names of the repository/repositories and accession number(s) can be found below: NCBI BioProject repository, https://www.ncbi.nlm.nih.gov/bioproject, PRJNA906646.

## Author contributions

MA, YL, HZ, and MH performed the experiments. QX and QC contributed to conception and design of the study. MM, KZ, and QX analyzed the data. MA organized the database and prepared the figures and tables. MA, YL, and HZ wrote the first draft of the manuscript. QX, YG, XY, MM, LZ, and QC wrote sections of the manuscript. All authors contributed to manuscript revision, read, and approved the submitted version.

## Footnotes

1
https://github.com/tanghaibao/goatools

2
http://kobas.cbi.pku.edu.cn/home.do

3
https://www.ncbi.nlm.nih.gov/tools/primer-blast/

4
www.cominbio.com
