# Supplementary material for: Transcriptomic profiling revealed important roles of amino acid metabolism in fruiting body formation at different ripening times in Hypsizygus marmoreus
Source: Front Microbiol. 2023 Apr 25;14:1169881. doi: 10.3389/fmicb.2023.1169881 (PMC10167310; doi:10.3389/fmicb.2023.1169881)
Supplement: Supplementary file 4 [file Data_Sheet_1.docx]

Supplementary Material

**Transcriptomic profiling revealed important roles of amino acid metabolism in fruiting body formation at different ripening times in *Hypsizygus marmoreus***

Quanju Xiang^1¶^, Arshad Muhammad^1¶^, Yakun Li^1¶^, Huijuan Zhang, YunfuGu^1^, Xiumei Yu^1^, Ke Zhao^1^, Menggen Ma^1^, Lingzi Zhang^1^, Maolan He ^2^ and Qiang Chen^1^*

*** Correspondence:** Qiang Chen: [cqiang@sicau.edu.cn](mailto:cqiang@sicau.edu.cn)

# Supplementary Tables

Table S1 Differentially expressed genes

Table S2 GO categories of differentially expressed genes.

Table S3 KEGG pathways of differentially expressed genes.

# Supplementary Figures


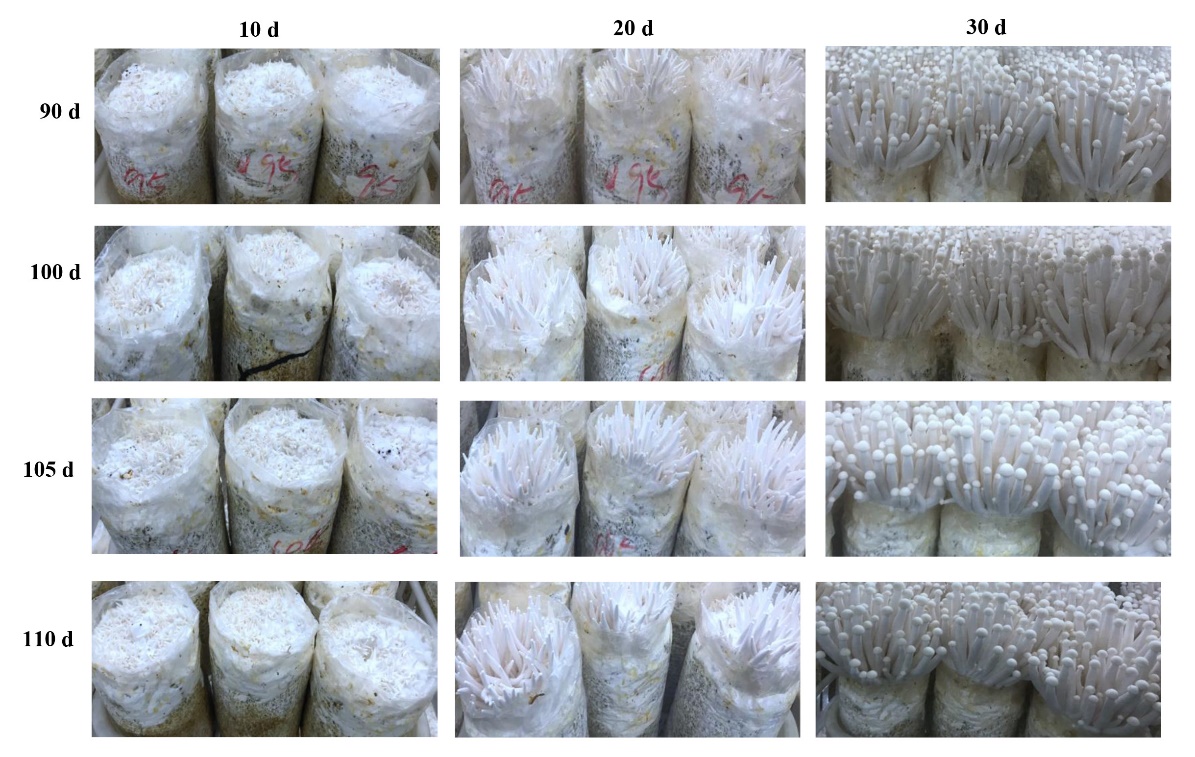


**Supplementary Figure 1.** The fruiting body from four ripening times.

The time on the left side indicated different ripening times, and the time on the top is the time after transformation to the fruiting room.
